# Role of Host and Pathogen-Derived MicroRNAs in Immune Regulation During Infectious and Inflammatory Diseases

**DOI:** 10.3389/fimmu.2019.03081

**Published:** 2020-01-24

**Authors:** Kumari Chandan, Meenakshi Gupta, Maryam Sarwat

**Affiliations:** Amity Institute of Pharmacy, Amity University, Noida, India

**Keywords:** miRNA, inflammation, infection, immunity, pathogen, host

## Abstract

MicroRNAs (miRNAs, miRs) are short, endogenously initiated, non-coding RNAs that bind to target mRNAs, leading to the degradation or translational suppression of respective mRNAs. They have been reported as key players in physiological processes like differentiation, cellular proliferation, development, and apoptosis. They have gained importance as gene expression regulators in the immune system. They control antibody production and release various inflammatory mediators. Abnormal expression and functioning of miRNA in the immune system is linked to various diseases like inflammatory disorders, allergic diseases, cancers etc. As compared to the average human genome, miRNA targets the genes of immune system quite differently. miRNA appeared to regulate the responses related to both acquired and innate immunity of the humans. Several miRNAs importantly regulate the transcription and even, dysregulation of inflammation-related mediators. Many miRNAs are either upregulated or downregulated in various inflammatory and infectious diseases. Hence, modifying or targeting the expression of miRNAs might serve as a novel strategy for the diagnosis, prevention, and treatment of various inflammatory and infectious conditions.

## Introduction

Inflammation is a protective mechanism of the body against any infection or tissue damage. The biological purpose of inflammation is to restore tissue homeostasis and to manage deregulation occurring during any sort of injury (1). According to Cornelius Celsus, inflammation can be characterized by pain (dolor), heat (calor), redness (rubor) and swelling (tumor). Physiologically, white blood cells (WBCs) and plasma components infiltrate from dilated vessels and migrate to injured tissues (2, 3). These inflammatory cells, not only act as a defense to the host body against the invasion of pathogens via phagocytosis but also, maintains tissue homeostasis once the danger signal has been abolished (4). However, chronic inflammation leads to a plethora of diseases including autoimmune and metabolic disorders (5). Persistent inflammation is not only related to typical inflammatory diseases but is also an essential feature in the pathogenesis of diseases like atherosclerosis, cardiovascular disease, Alzheimer's disease, and cancer (6, 7).

Our body has a rich variety of flora and fauna, which helps in digestion, pH maintenance and development of the immune system (8). However, exogenous pathogenic microorganisms like bacteria, viruses, fungi, and parasites can cause disorders termed as infectious diseases (9). These exogenic pathogens either disrupt the normal physiological processes or modulate the responses of the immune system resulting in high fever and inflammation. These pathogens mainly enter into the human body via a vector or through contact with body fluids. Successful entry of pathogens to the host body leads to invasion of host immune response by pathogen and its replication and dissemination to the host cell and tissues. A large number of bacterial, viral and fungal species have been reported to be pathogenic, which overcome host immune defenses, invade the tissues and cause various infectious diseases (10, 11). Molecular pathways that regulate the extent of inflammatory and infectious responses have been discovered. miRNAs play important role in these pathways (12). miRNAs are generally small (20–22 nt) non-coding parts of RNA accounting for about 1–2% of mammalian genes. They act by binding to the targeted mRNAs, which either gets degraded or translationally inhibited. It was first reported in a nematode *C. elegans* in 1993, where it was seen that miRNA lin-4 regulates the expression of gene lin-14. Researchers have reported that the expression of multiple genes can be regulated by a single miRNA (13–15). miRNAs are important in the survival and functioning of various immune cell types and have been reported to play an important role in mediating responses to infections. This property of miRNAs make them potential candidates for the management of immunity as well as controlling infectious diseases (16).

Pathogens encoded miRNA is utilized for the survival and multiplication of pathogens in the host body. These microorganisms either interfere with various physiological processes for their survival during infection or alter the host machinery for their own benefit by changing the pattern of miRNA expression (17). Several reports are present in literature that showed the influence of miRNA in various infections. Kincaid and collaborators discovered miRNAs from a bovine leukemia virus (BLV) possessing RNA as genetic material (17). The viral encoded miRNAs promote viral replication and control latency. These viruses use host cell machinery to make their own miRNA. These miRNAs downregulate the factors promoting the inflammatory response of the host (18–20). The herpes simplex virus type 1 (HSV-1) gene encodes miRNA-H2-3p, which promotes the replication of HSV-1 and reactivation from latency. Similarly, the response of CD8+ T-cells has been inhibited by Cytomegalovirus (CMV) via expression of miRNA-US4-1 targeting endoplasmic aminopeptidase-1 (a protein responsible for trimming peptides for presentation by major histocompatibility complex (MHC) class I molecules) (20). miRNA-K5 and miRNA-K9 associated with Kaposi's sarcoma target myeloid differentiation primary response gene-88 (MyD88) and Interleukin 1 receptor-associated kinase 1 (IRAK1), which further reduces the expression of inflammatory cytokine and clearance by the immune system (21). Cai and their team explored the Japanese encephalitis virus (JEV)- infected PK15 cell line and found upregulated and downregulated miRs specific for the infection. Sharma and colleagues studied JEV infected human microglial cell line CHME3 and found that miRNA-146a targets the cytokine signaling system via transcriptional downregulation of IRAK1 and TNF Receptor Associated Factor 6 (TRAF6). Additionally, miRNAs of the immune system are reported to have important roles in signaling, differentiation, or pathogenic defense (17). In this review, we have summarized the role of host- and pathogen-derived miRNAs in immune regulation during infectious and inflammatory diseases.

## Biosynthesis of miRNA

MicroRNA is synthesized by enzymes called RNA polymerase II and III. Primary miRNA (pri-miRNA), formed after transcription, is processed to form precursor miRNA (pre-miRNA) in the presence of microprocessor multi-protein complex, and the co-factor DiGeorge syndrome Critical Region 8 (DGCR8/Pasha) (22). This complex is exported to the cytoplasm from the nucleus by exportin 5 (XPO5). XPO5 is a 22 nucleotide duplex, designed by RNAse type III enzyme- Dicer. Dicer along with Trans-Activation responsive RNA-binding protein 2 (TARBP2) and Argonaute (AGO) family proteins form a complex, which further triggers the association of RNA-induced silencing complex (RISC). One strand of miRNA is degraded and the other strand ushers the RISC to the target mRNA through base pairing. Although both strands are functional, only one strand is used. The identification of the target site by miRNA depends upon the seed sequence (conserved heptameric sequence) of the miRNA (23).

## Role of miRNAs in Immune Regulation

**Figure 1:**
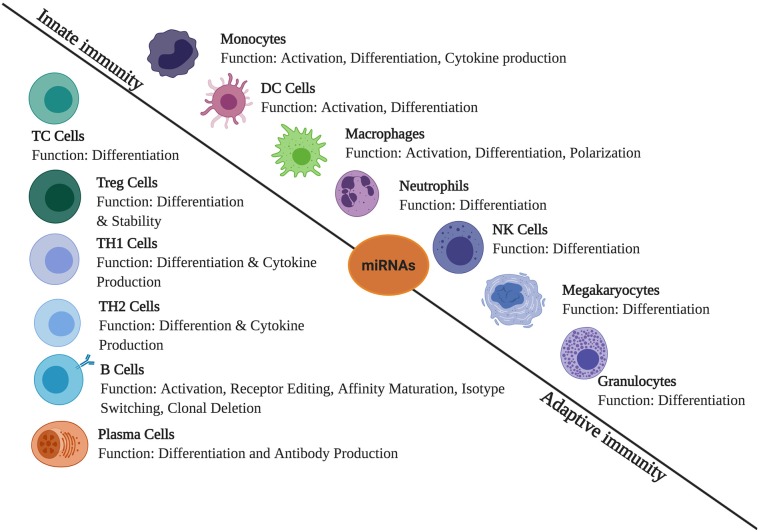
The role of miRNAs in immune regulation. They are expressed in immune cells and play a role in the regulation of innate and adaptive immune responses. They create regulatory networks in innate immunity, regulate functions of immune cells such as monocytes, dendritic cells (DCs), macrophages, neutrophils, natural killer (NK) cells, megakaryocytes, and granulocytes. In adaptive immunity, they also regulate immune signaling pathways involved in the T- and B-cell development, differentiation, central and peripheral tolerance, as well as their function.

Massive reports have been published which states the role of miRNA in regulating immunological responses including development, maturation, activation, functioning, and aging of various immune cells (Figure 1). It has been observed that several miRNAs exhibit highly specific expression patterns of organs associated with the immune system. Even the differentiation of hematopoietic progenitor cells into either the lymphoid or myeloid lineage is modulated by the expression profile of various miRNAs. This clearly suggests a significant role of miRNAs in immune cell development and functioning (24). Both innate and adaptive immune responses are influenced by miRNAs, leading to their impact on the outcome of a variety of diseases. Therefore, it is necessary to understand how miRNAs regulate different physiological processes of the immune system in the normal and diseased state (25).

### miRNAs as Regulators of Innate Immune Response

Innate immune response acts as a primary line of defense against foreign pathogens and is considered as the spark plug of various inflammatory responses. These responses are originated by pattern recognition receptors (PRRs) that respond to pathogen-associated molecular patterns (PAMPs) (26). PRRs are classified into five classes: C-type lectin receptors (CLRs), Nod-like receptors (NLRs), RIG-like receptors (RLRs), AIM2- like receptors (ALR) and Toll-like receptors (TLRs). They play an important role in regulating the innate immune response. Particularly, TLRs usually mediate the identification of a variety of pathogens (27). TLR3 recognizes double-stranded RNA present in viruses whereas TLR4 is for identifying bacterial products, especially ligand lipopolysaccharide (LPS). Recognition of pathogens by TLRs triggers various intercellular signaling. This cascade is further divided into two pathways: MyD88 and Toll/IL-1R domain-containing adaptor-inducing IFN-β (TRIF) dependent pathway. MyD88 is used by TLR2, TLR5, TLR7, TLR8, and TLR9; TRIF is used by TLR3 wheres TLR4 utilizes both these pathways for the activation of pro-inflammatory cytokines and interferon (IFN) stimulated gene (28). Both these pathways have been reported to trigger Nuclear Factor Kappa B (NF-κB) signaling, a crucial regulator of the innate immune response. NF- κB is targeted by a number of miRNAs and these are found to modulate an innate immune response. Stimulation of these pathways also activates other cascades like mitogen-activated protein kinase (MAPK) and interferon regulatory factor 3 (IRF3) (29). Recent literature evidences the regulation of acute inflammatory response by several miRNAs, namely miRNA-155, miRNA-146, and miRNA-223 subsequent to recognition pathogen (30).

#### Regulation of miRNA of Macrophage and Monocytes

Macrophages and monocytes are implicated in the regulation of inflammatory and infectious diseases. These cells, when exposed to any inflammatory/infectious stimuli, initiate the production of cytokines. Additionally, microbial phagocytosis is observed during innate responses by these cells (31, 32). It has been highlighted that miRNA can affect and modify all stages of the macrophage life cycle (starting from production to differentiation) (32). An experiment by O'Connell and colleagues in 2007 identified the changes induced in miRNA-155 of primary murine macrophages when they were exposed to polyriboinosinic: polyribocytidylic acid [poly (I: C)] or cytokines IFN-β. They have demonstrated that miRNA-155 can be activated/regulated by TLR ligands. In this experiment, macrophages were stimulated with LPS, which signals through TLR4/TLR9/ hypomethylated DNA or Pam3CSK4, a synthetic activator of TLR2 (33). Tili and collaborators in 2007 observed miRNA-155 targets the transcript coding proteins of TNF-α gene, which led to TNF-α stimulation. miRNA-155 acted by targeting SH-2 containing inositol 5′ polyphosphatase 1 (SHIP1) directly, leading to increase activation of Akt kinase thus, driving the inflammatory response. This provides evidence for both positive and negative post-transcriptional activity of miRNA-155 via regulation of target proteins such as Fas-associated death domain (FADD), IκB kinase ε (IKKε) and receptor-interacting serine-threonine kinase 1(Ripk1) (33).

Another miRNA, miRNA-146 worked by regulating the NF- κB pathway, directing the expression of IRAK1 and TRAF6 in macrophages. IRAK1 and TRAF6 are adaptor molecules of the MyD88-dependent signaling pathway. Stimulation of TLR stimulated activator protein 1 (AP-1) and NF-κB transcription factors mediated various immune responses (34). Type-I IFN production initiated by vesicular stomatitis virus was also negatively inhibited by the expression of miRNA-146a through the RIG-1 pathway. The possible targets reported are IRAK1, IRAK2, and TRAF6 in macrophages. Hence, during innate responses, miRNA−146a negatively regulate TLR and cytokine signaling. Similarly, miRNA-9 induction by TLR and cytokines activation in monocytes controlled NF-kB dependent responses. miRNA-125 has been found to repress mRNA transcripts of TNF- α and is downregulated by the action of LPS (29, 34). In macrophages, the excessive inflammatory response is inhibited by miR-147 via activation of TLR2, TLR3, and TLR4 in both MyD88 and TRIF dependent manner (34). During RNA virus infection, miR-21 has been reported to inhibit MyD88 and IRAK1 expression, which further, upregulated JNK/c-Jun signaling pathway, ERK/c-Fos pathway and interferon signaling pathway (35). During the process of monocyte-macrophage differentiation, decreased expression of miRNA-223 has been observed. This decreased expression increased the expression of IKKα, a serine-threonine kinase, in human monocytes and macrophages. Higher expression of IKKα along with stabilization of the kinase NIK elevated p52, which resulted in the repression of NF-κB target genes (36). Another miR, miR-92a, is found to target MAKK4, inhibiting the inflammatory response triggered by TRL4 in macrophages (37).

#### miRNA Regulation of Granulocytes

MiRNAs play an essential role in the proliferation and functioning of granulocytes. In particular, miRNA-223 and miRNA-155 have been brought into the highlight. Both negative and positive regulation of miRNA-223 have been observed by the researchers. Fazi and colloborators in 2005 displayed that granulocyte differentiation is positively regulated by miRNA-223. The activity of miRNA-223 expression is mediated by nuclear factor-IA (NF-IA) and the CCAAT enhancer proteins (C/EBPa). NF-IA is necessary to maintain low levels of miR-223 but while differentiation, it is substituted by C/EBPa leading to increased expression of miRNA-223 (38). Knockout of miR-223 show its negative role in regulating differentiation and activation of granulocyte by directly targeting Mef2c, (a transcription factor necessary for myeloid progenitor proliferation). miRNA-223 has also been found to control cell functions in human neutrophils induced by LPS via regulation of MYD88/NF-κB. Another miR, miRNA-155 has been found to act on SHIP1 in granulocyte or macrophage expansion during inflammation (39).

#### miRNA Regulation of Dendritic Cells

Dendritic cells (DCs) are antigen-presenting cells originated from hematopoietic progenitor cells and circulating monocytes. They promote an immune response to exogenous bodies and maintains self-tolerance. These cells serve as a bridge between innate and adaptive immune systems of our body (40). Providing co-stimulation and cytokines necessary for T-cell activation is also a function of DCs. They express a variety of pathogen recognition receptors like TLRs to initiate their maturation and migration to the lymph nodes. In cells of the innate and adaptive immune system, the expression of more than 100 miRNAs have been reported. miR-21 and miRNA-34 inhibitors stalled monocyte-derived dendritic cells (MDDC) differentiation in monocytes, an effect that was boosted upon inhibition of both miRNAs. These miRs regulated the expression of the Wnt Family Member 1 (*WNT1*) and Jagged Canonical Notch Ligand 1 (*JAG1*) gene. Importantly, during MDDC differentiation, the expression of mRNAs of *WNT1* and *JAG1* was found to be increased (41). Naturally, DCs express low levels of miR-146a but during the differentiation of DCs, the expression of miR-146a was upregulated using granulocyte/macrophage colony-stimulating factor (GM-CSF) and IL-4. It modulate the production of pro-inflammatory cytokines in mature DCs. This miRNA also targets TRAF6 and IRAK-1 in these cells and therefore, increased the apoptosis of DCs (42). miRNA-155 is important for the functioning of dendritic cells. It increase the pathogen uptake of DCs by downregulating DC-SIGN via repression of PU.1 expression. It also modulate IL-1 signaling by regulating levels of TAB2, which decreases the production of cytokines in activated human MDDCs (42). These reports shows that miRNAs play important roles in modulating DC function in both types of immune responses.

### miRNA Regulation of Adaptive Immunity Response

Adaptive immune responses are majorly characterized by activation and clonal expansion of T- and B-cells. This activation and expansion lead to cytotoxic effector response and the production of antibodies in response to infections (43). miRNA has been widely associated with modulating adaptive immunity by regulating the development, activation, survival, and proliferation of T- and B-cells (25).

#### miRNAs Regulation of T-Cell Differentiation and Activation

T-cells are responsible for specific inflammatory responses. This activity is mediated by the presence of specific antigens in the context of MHC (44). The development of T-cells involves the role of various signaling cascades, mediated by miRNAs. The significance of miRNAs in controlling functions of T-cells is revealed in miRNA-deficient mice. Additionally, few expressed miRNAs were observed while profiling effector and memory CD8+ cells. Increasing levels of CD8+ T-cells are found to be linked with the downregulation of miRNAs in effector CD8+ T-cells (45). Also, it has been observed that miRNAs with shorter 3′UTRs are expressed more during the activation of CD4+ T- cells. A large number of studies put forward the potential of miRNAs during the activation and differentiation of T-cells. Disruption in the biogenesis of miRNA can cause conditional removal of dicer in the early developmental stage, resulting in a reduced T-cell count. Additionally, deviant differentiation of T-helper cells and cytokine production, along with decreased survival of αβ-expressing thymocytes are also observed. Reduction in the number, poor proliferation, and increased apoptosis are also observed in peripheral T-cells (46, 47). Certain dynamic changes are observed in the expression pattern of miRNAs in the T-cell subsets. Differential expression of various miRNAs, namely, miRNA-181 family, miRNA-17-92 clusters, miRNA-214, miRNA-146a, miRNA-155, let-7, miRNA-29, miRNA-125, and miRNA-216 has been observed in the signaling cascade downstream of T-cell activation (48). Upregulation of miRNA-181 affect multiple targets, including SH2 domain-containing protein tyrosine phosphatase 2 (SHP2), protein tyrosine phosphatase non-receptor type 22 (PTPN22), dual-specificity protein phosphatase 5 (DUSPS5) and DUDPS6, to heighten TCR signaling. This enhances the phosphorylation of the activation site of the cytosolic TCR/CD3 complex. Expression of miR-181 leads to the deletion of some T-cell clones, allowing the maintenance of central tolerance (49). Similarly, miRNA-155 has been observed to play an essential role in CD4+ T-cell differentiation. The overexpression of this miRNA is associated with the differentiation of these cells into Th1 cells and reduced expression show a bias toward cell differentiation (50). Another miR, miRNA-17-92 enhanced the production of IFN- γ and suppressed the differentiation of regulatory T (Treg) cells while promoting Th1differentiation (51). miRNA-326 targets ETS-1 (negative regulator of Th17) promotes Th17 differentiation and development its overexpression is linked to production of IL-17 (52). miRNAs have also been confirmed to upregulate miRNA-214 and miRNA-17-92 cluster, promoting the activation and proliferation of T-cells by targeting phosphatase and tensin homolog (PTEN) in the PI3K/Akt strain transforming (AKT) pathway (48). Another miRNA, miRNA-146 modulates the response of the immune system by targeting TRAF6 and IRAK1 of the NF- κB signaling in activated T-cells (52).

In addition, miRNAs play a critical role in regulating Treg cells function. Treg cells are required for maintaining immune cell homeostasis by limiting immune responses and preventing autoimmunity. Deletion of dicer in forkhead box protein P3+ (Foxp3+), mainly due to deficiency of miRNAs, also induce fatal auto-immune pathologies. Among various miRNAs, miRNA-10a, miRNA-146a, and miRNA-155 have contributed to Treg homeostasis and functions (53).

miRNA-10a restricts the transformation of Treg cells into T-follicular helper cells by acting on transcriptional repressor Bcl-6, hence contributing to the stability of the Treg cell phenotype. Induction of Treg occurs by TGF-β and retinoic acid is during inducible Treg cell differentiation from CD4+ T-cells. Retinoic acid is stimulated by TGF-β, which upon stimulation regulates miRNA-10 expression (54). miRNA-10a directly targets Bcl-6, and nuclear receptor corepressor 2 (Ncor2), which co-represses RARα, initiating positive feedback. Downregulation of Bcl-6 by miRNA-10a, has also been reported this elicits higher levels of T-cell specific T-box transcription factor (Tbet), which is a known inhibitor of Th17 differentiation (55). miRNA-17-92 increases T-cell survival during development by suppressing the expression of pro-apoptotic proteins (including BIM and BCL2L11) and PTEN (51).

#### miRNAs Regulation of B-Cell Development, Differentiation, and Activation

B-cells begin to develop in primary lymphoid tissue and mature in secondary lymphoid tissues. These cells are responsible for the antibody-mediated response. Recent literature highlights the role of various miRNAs in the development and differentiation of B-cells. Expression of miRNA in naive, germinal central (GC) and post-germinal central B-cells has been observed, implying the role of miRNAs in the development and maturation of B-cells (6). Xu and co-workers in 2012 have highlighted the role of miRNA in B-cell differentiation. A hematopoietic defect in Ago2 has not only affect early pro-B-cell generation but also significantly impairs pre- and peripheral B-cell generation. Also, the deletion of dicer or Ago2 blocks the transition of pro-B to pre-B-cell development, underscoring the role of miRNAs (56, 57).

B-cell population reducts has been observed due to the conditional deletion of dicer. The possible target is C-Myb, which is critically important in B-cell development. The expression pattern of C-Myb was inversely correlated to the expression of miRNA-150 in B-cells. Higher expression of miRNA-150 is observed in progenitor cells whereas mature B-cells represent its downregulation. Ectopic expression of miRNA-150 followed the premature downregulation of C-Myb, trigger apoptosis during the pro-B stage. Higher levels of miRNA-150 are necessary for the conversion of pre-B to mature B-cells (to downregulate C-Myb expression), guaranteeing normal B-cell development (58). The expression of miRNA-34a block the conversion of pro-B to the pre-B-cell. This blocking is probably mediated by miRNA-34a, which inhibits the expression of forkhead box transcription factor, Foxp1. The loss of Foxp1 resulted in a severe blockade in the development of B-cells (59). In naïve CD4 positive cells, miRNA-155 represses the expression of c-MAF (transcription factors) and IFNγ receptor 1, whereas, in B-cells, it blocks the expression of PU.1 and SHIP1. The absence of miRNA−155 produces defected antibodies and hence, impaired response to antigenic stimulation. Murine model deficient in miRNA-155 show an increased number of GC B-cell, decrease IgG production and maturation. This regulation of GC cells is mediated via regulation of transcription factor PU.1 at the post-transcriptional level (59).

The list of miRNAs involved in the development and function of the immune system is provided in Table 1 (60–68).

**Table 1:** miRNAs Involved in the Development and Function of the Immune System.

**miRNA**	**Target**	**Function**	**References**
miR-181	SHP2, PTPN22, DUSPS5	Enhance TCR signaling and enhances the phosphorylation of immunoreceptor	(49)
	Ox-LDL	Increased DC maturation	(60)
miR-155	SHIP1	Increasing activation of the kinase Akt, which drives the inflammatory response	(33)
	PU.1	Decreased production of cytokine	(59)
	CTLA-4	differentiation and activation of Th cells and effectively inhibit inflammation	(61)
	MMP-1	Induces proinflammatory cytokines and activation of TLR ligands	(62)
Mir-10a	TGF-β	Treg cell differentiation from CD4+ T- cells	(54)
	IL-12/IL-23p40, NOD	Decrease mucosal inflammatory response and inhibiting Th1 and Th17 cell function	(63)
	TAK1, IL-1, β-TrCP	Inhibitor κB (IκB) degradation and NF-κB activation	(64)
miR-145	SMAD3	Inhibits the release of IL-6 and CXCL8 in chronic pulmonary disease	(64)
miR-21	RASGRP1	Control T cell activation and induced T cell receptor (TCR)	(65)
	PDCD4	Elevated production of IL-10, regulate T-cell response and negatively regulate the inflammatory response to LPS	(66)
miR-146	STAT1	Th1 effector cell differentiation, and suppress Th1 responses	(67)
	IRAK1, TRAF6	Negative regulator of the IFN pathway and immune response, reduce inflammatory cytokine production	(17)
	(AP)-1, IL-2	Immune cell activation and cytokines production, a negative regulator of adaptive immunity	(34)
MiR-29	IFN-γ	Suppress immune response	(68)

## miRNAs Immune Regulation In Inflammatory Disease

**Figure 2:**
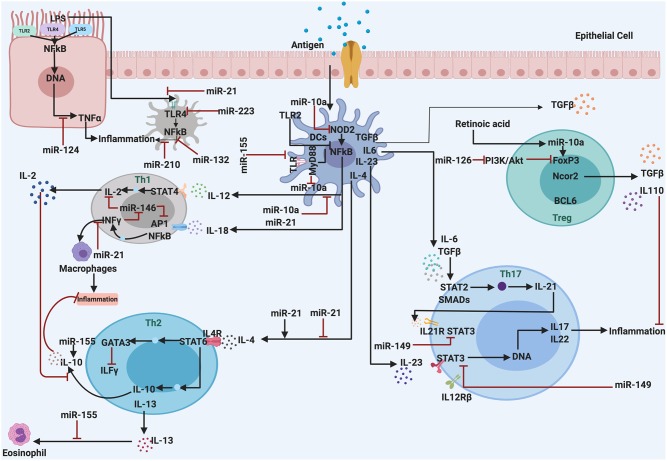
The miRNAs are associated with regulation and control the inflammatory response. They act as anti-inflammatory miRNAs, serve in important negative feedback loops in inflammation processes and inflammatory diseases. By targeting signal transduction proteins involved in the initiation of the innate immune response, and by directly targeting mRNAs that encode specific inflammatory mediators.

The role of miRNA in regulating the responses of the immune system and inflammatory processes has been reported a few years ago. Recent literature highlights the association of miRNAs and inflammatory processes in various metabolic disorders. Proteins involved in inflammatory processes can be regulated by miRNAs at the transcriptional level (69, 70). Also, initiation of inflammation and other physiological responses like oxidative stress, adipogenesis, and macrophage activation are regulated by miRNAs (Figure 2). Therefore, the deregulation of miRNAs linked to the immune system may lead to chronic inflammation, which is a hallmark of sustained inflammatory diseases (71). Some detailed examples of inflammatory disease that are regulated by miRNAs are discussed as follows.

### Psoriasis and Atopic Eczema

Psoriasis and atopic dermatitis (AD) are chronic inflammatory skin diseases that are described by various immunological abnormalities. Psoriasis is mainly characterized by Th1/Th17 response whereas patients suffering from AD have shown an imbalance of T-cells and dysfunction in the cytokine and chemokine synthesis (72). Various miRNAs have been shown to be involved in the pathogenesis of psoriasis and atopic dermatitis (61, 73). In a study conducted by Fu and coworkers it is observed that decreased expression of miRNA-138 in CD4+ T-cells increases the expression of runt-related transcription factor 3 (RUNX3) and the ratio of Th1/Th2 in the cells obtained from patients suffering from psoriasis. Overexpression of miRNA-31 targets SKT40 (a negative regulator of NF-κB), and modulates the production of IL-1β, CXCL1/5/8, and IL-8 in *in vitro* and *in vivo* models of psoriasis (74). Excessive expression of miRNA-203 and miRNA-146a inhibited the suppressor of cytokine signaling-3 (SOCS-3) (cytokine signaling 3), which is followed by increased proliferation of keratinocytes. Deregulation in the expression of miR-146a affect the functioning of T-helper and dendritic cells, increasing the severity of lesions by increased TNF-α production (73). Similarly, the literature reflect the differential expression profile of miRNA-22, miRNA-24-1, miRNA-498, and miRNA-551a in the skin of healthy and affected patients (73). miRNA-155 is observed to be highly expressed in the tissue samples obtained from patients suffering from AD. This expression is initiated by exposure of allergens or super-antigens and is highly specific during Th cell differentiation and activation. miR-155 has been reported to alleviate the expression of cytotoxic T-lymphocyte associated antigen- 4 (CTLA-4), which leads to increased Th cell response. CTLA-4 is a key player in inhibiting T-cell response and its expression is suppressed by miR-155 in Th cells (61). Other miRNAs that are reported in the tissue samples obtained from AD patients are miRNA-146a, miRNA-10b, miR10a, miRNA-10a*, miRNA-216, miRNA-921-1*, miRNA-454 and miRNA-29b-1*(upregulated); and miRNA-99a*, miRNA-34a*, miRNA34c-5p and miRNA-30a (downregulated) (61).

### Asthma

Asthma is a chronic respiratory disease of the airways characterized by up-modulated expression of inflammatory proteins, at the molecular level (75). The proliferation of smooth muscle and epithelial cells of lungs are the hallmarks in asthma. In both these cell types, the role of miRNAs has been widely observed. The expression of miRNA-221 is found to be elevated in the smooth muscle cells in asthmatic patients (76). miRNA-155 has a key role in the development and maintenance of the Th2 phenotype via modulation of IL-13. IL-13 (Th2 derived effector cytokine) is an essential factor in the pathogenesis of asthma and it induces allergic airway inflammation. Inhibition of miRNA-155 increases the levels of transcription factors involved in the Th2 microenvironment (77). The upregulation of miR-19a in asthma patients promotes the *in vitro* cytokines production. This miRNA acts on the mRNA that encodes PTEN, SOCS1, and A20, which collectively suppresses several physiological cascades (76). The expression of IL-12p35 is influenced by miRNA-21 in macrophages and dendritic cells. IL-12 is centrally involved in the polarization of Th1 cells in the adaptive immune response. This shows that miR-21 mediate expression of IL-12p35 potentially regulates the Th1/Th2 balance (78). In patients with severe asthma, TGF-β induces the expression of miRNA-122 in the airway smooth muscle cells. Further, this miRNA increases the secretion of IL-6 (79).

### Rheumatoid Arthritis

Rheumatoid arthritis (RA) an is irreversible joint damage, which occurs due to inflammation of synovial tissue. T- and B-cells, monocyte/macrophages, neutrophils, and proliferating synovial fibroblast-like cells are the major contributors to the cellular damage in RA (80). Various miRNAs like miRNA-203, miRNA-16, miRNA-146a, miRNA-124a, miRNA-155, miRNA-15a, and miRNA-346 have been recognized in RA tissues (81). Stanczyk and colleagues in 2008 have identified TNF-α regulated miRNA-155 in synovial fibroblasts of RA patients. Overexpression of miRNA-155 in RA tissue induces proinflammatory cytokines and activation of TLR ligands via suppression of metalloproteinases MMP-1 and 3 (62, 82). miRNA-146a modulates MMP-13 by acting onIRAK1 and TRAF6. Studies have demonstrated its active role in T-cell apoptosis and in suppressing functions of Treg cells (83). Expression of miRNA-346 induces the secretion of MMP1 and IL-6 (by NF-kβ signaling) and suppresses IL-18 response (by inhibition of Bruton's tyrosine kinase gene transcription). Additionally, the release of TNFα is also controlled by miRNA-346 in RA tissue (84).

### Chronic Obstructive Pulmonary Disorder (COPD)

Chronic and systemic inflammation, along with defective immune response is the major characteristic of chronic obstructive pulmonary disorder (COPD) (85). Innate immune cells sense the destruction in the lungs by damage-associated molecular pattern/ pattern recognition receptor (DAMP/PRR) signaling and hence, an adequate immune response is initiated (85, 86). The role of various miRNAs (miR-1, miR-21, miR-144, miR-145, miR-146, and miR-181) in the COPD pathogenesis has been reflected in the literature (86). For example, miR-145 targetsSMAD3, which is an important regulator of TGF- β pathway. miR-145 also regulates p38 and MAPK pathways. The upregulation of this miR significantly inhibits the release of IL-6 and CXCL8. Similarly, miR-146 reflected a pathogenic role in cultured fibroblasts of COPD patients by regulating NF-κB signaling (87).

### Atherosclerosis

Atherosclerosis (AS) affects major arteries of the body, which may result in myocardial infarction, ischemic stroke, and peripheral artery disease (88). The expression profiles of various miRs such as miRNA-21, miRNA-34a, miRNA-146a, miRNA-146b-5p, and miRNA-210 have shown significant upregulation. These miRNAs act on multiple targets in the human atherosclerotic plaques. miRNA-146 (anti-inflammatory) and miRNA-145 (proinflammatory) act by regulating dendritic cell functions while, other like, miRNA-155 stimulates inflammatory mediators (Nos2 and IL-6) through inhibition of Akt1 pathway in macrophages. MiR-155 also acts on MAP3 K10 of the MAP kinase signaling in the patients suffering from coronary artery disease (CAD) (88, 89). Endothelial progenitor cells of CAD patients show higher expression of miRNA-221 and miRNA-222. miRNA-10a expression in endothelial cells is decreased in atheroprone areas of the porcine aorta as compared to atheroprotective regions, indicating that this miRNA potentially behaves as an anti-atherogenic miRNA. Analysis of the functional activity of miRNA-10a revealed that it exhibits potent anti-inflammatory properties, mediated by the inhibition of NF-κB activity (90).

### Inflammatory Bowel Disease (IBD)

Ulcerative colitis and Crohn's disease are collectively termed as inflammatory bowel disease (IBD) (91). The major factors that contribute to the pathogenesis of IBD include alterations in the immune component, genetic material, bacteria, and environment. Increased incidences of IBD are reported in the developed countries in the last few decades, which clearly states the role of various environmental and epigenetic factors in the pathogenesis of IBD (92, 93). Also, excessive filtration of immune cells and tissue damage has been widely observed in IBD patients. These alterations in the architecture of cells and tissue may be due to the production of cytokines (IL-1) family and chemokines (92). The possible relevance of Th cells has also been reported in the pathogenesis of IBD. Th cells act by releasing IL-17, a strong pro-inflammatory factor. Studies have shown how miRNAs regulate the inflammatory response in IBD (94). miRNA-21 is significantly increased in the fibroblasts of active UC. This miRNA is associated with nitric oxide synthase (*NOS2*) and CD68 in IBD. It increased concentration of nitric oxide (NO) and the activation of macrophages. Yang and colleagues in 2017 have demonstrated the impairment of intestinal epithelial function in UC patients. The possible mechanism may be the overexpression of miRNA-21 via RhoB signaling (93). Additionally, the regulation of *NOS2* by miRNA-221 miRNA-146a, miRNA-223, and, miRNA-126 in IBD tissue has also been reported (95). In HCT116 cells, the suppression of *NOD2* is mediated by miRNA-671, miRNA-495, miRNA-192, and miRNA-512. The expression of miRNA-10a inhibits IL-12/IL-23p40 and *NOD2*, decreasing mucosal inflammatory responses in DC (96). miRNA-146a, on the other hand, targets the NUMB gene, modulating sonic hedgehog (SHH) in macrophages of the murine model. Reportedly, miR-29 mediates the effect in both, direct (encodes IL-12/23 p40) and indirect (suppression of transcription factor ATF2 of IL-23A) manner (93).

The list of miRNAs Involved in the inflammatory process and their target genes are shown in Table 2 (97–110).

**Table 2:** Role of miRNAs in the inflammatory process and their target genes.

**miRNA**	**Target gene**	**Cell type or tissue**	**Effect**	**References**
miR-132	NF-κB	*In vitro* differentiated adipocytes and human adipose-derived stem cells	Overexpression induces translocation of NF-κB, acetylation of p65, and production of IL-8 and MCP-1.	(97)
	IL-6	Human adipose tissue	Related to macrophage infiltration and IL-6levels in patients suffering from nonalcoholic. Fatty liver disease.	(97)
miR-126	VCAM-1	Human aortic endothelial cells	Increased expression is observed in response to anti-atherogenic triglyceride-rich lipoproteins or polyunsaturated fatty acids treatment.	(98)
miR-145	TNF-α	Human *in vitro* differentiated adipocytes	Increases the release/production of TNF-α.	(99)
miR-146	IL-1β	Primary human gingival fibroblasts in culture	IRAK inhibits mir-146 leading to upregulation of IL-1 and inhibits inflammatory response in periodontal inflammation.	(100)
	TNF-α	Human monocytic cell line THP-1	miR-146 is NF-κB dependent and acts as an inhibitor targeted to signaling proteins of innate immune responses.	(101)
miR-181	NF-κB/VCAM-1/ E-selectin	Human plasma	Overexpression inhibits import in a3 expression and an enriched set of NF-κB-responsive genes.	(102)
miR-187	TNF-α, IL-6, and IL-12	TLR4-stimulated monocytes	Regulates cytokine production.	(103)
miR-221	TNF-α	Human preadipocytes	Down-regulated by TNF-α.	(104)
miR-155	IL-1	Dendritic cell	During dendritic cell maturation, it regulates the TLR/IL-1 pathway.	(42)
miR-222	ICAM-1	Glioblastoma multiform tissue and colorectal cancer cells	Decreases the ICAM-1 expression and restricts the association of cytotoxic T lymphocyte cells to tumor cells.	(106)
miR-223	PAI-1	Monocytes	Avoids accumulation of NLRP3 protein and inhibits IL-1b production from the inflammasome.	(107)
Let-7	IL-6	Bone marrow–derived mesenchymal stem cells	Reduces expression of IL-6.	(108)
miR-24	Chitinase 3-like 1	Macrophages	Overexpression increases the production Arg1, CCL17, CCL22, CD163, and CD206 but reduces the production of phenotype markers in stimulated macrophages.	(109)
miR-124	TLRs	Monocytes and macrophages	Induces anti-inflammatory effects by downregulating TLR-6 and Myd88.	(110)

## miRNA Immune Regulation in Infectious Diseases

The immune system plays an important role in shielding the human body against invasion from infectious agents. Mostly, microorganisms are destroyed by the cells of the immune system but any deficit in the functioning of these cells directly associates with increased susceptibility to infections and diseases (111). During any pathogenic attack, the environmental and physiological conditions are altered in pathogens as well as the host body. The intracellular pathogen invades the machinery of the host and utilizes it for the expression of virulence genes. Similarly, the immune system of the host acts in a coordinated manner to combat microorganisms. Immune regulation in infectious diseases is mediated by both innate and adaptive immunity (112, 113). The cells of the innate system identify the conserved region on the pathogen. This activates complement, and targets them for phagocytosis. The phagocytic cells utilize reactive oxygen species, peptides and degrading enzymes and destroy the invading pathogen (114). Certain signaling molecules are also released by these cells to activate the responses of the adaptive immune system. The responses are produced in two ways: the cell-mediated response is carried out by T-cells and the humoral response is regulated by B-cells and antibodies. Interleukins and growth factors are produced which further regulate immune responses (115). The foreign invasion alters the expression profile and functioning of various miRNAs, which are directly involved in the pathogenesis of infections and diseases (17).

### Human Immunodeficiency Virus (HIV) Infection

HIV infection progresses to develop acquired immunodeficiency disease (AIDS), which is an immuno-compromised condition, suppressing cell-mediated immunity, HIV infected or uninfected decreased CD4+ T-cell count and weakening of the immune system. Although, monocytes/macrophages are reported to be susceptible to HIV-I infection, monocyte-derived macrophages are better producers of HIV-I when compared to MDDCs. Both these cells express various anti-HIV-1 miRNAs, modulation of which alters the cellular susceptibility to HIV-1 infection (116). miRNA-198 is downregulated during the differentiation of monocyte to macrophages. It inhibits the HIV-1 replication by downregulation cyclin T1 protein, demonstrating its anti-HIV function. miRNA-29a and miRNA-29b has been reported to inhibit Nef expression and HIV replication in HEK293T and Jurkat T-cells. Inhibition of miRNA-29a and miRNA-29b increases HIV-I production (117, 118).

HIV infected patients show different expression profiles of both host and viral miRNAs. Thus, miRNA can be utilized to distinguish between the individual with or without HIV infection (118). Gupta and team analyzed 704 categories of miRNAs in peripheral blood mononuclear cells (PBMCs) of HIV-1 infected and healthy volunteers. 28 miRNA are upregulated and 14 are downregulated in HIV patients (119). In particular, miRNA-150, miRNA-223, miRNA-191 and miRNA-146b-5p are downregulated and expressed excessively in T-cells. miRNA-223, miRNA-382, miRNA-125b and miRNA-28 target the 3′UTR region of HIV-1 mRNA and decrease HIV replication. miRNA-150 binds to Nef-3′ LTR at 773 and 89 positions, reducing the expression of PBMC opioid receptors. On the other hand, miRNA-223 acts on the 408th amino acid of HIV-1 protein. Interestingly, miRNA-17-5p and miRNA-20 downregulate p300/CBP associated factor (PCAF) histone acetyl-transferase expression, which inhibits the growth of HIV virus in the human body (113). Elevation of miRNA-122 and miRNA-21 is observed in patients suffering from HIV which is linked to the development of liver cirrhosis and pulmonary arterial hypertension by interfering with the TGF-β signaling (120).

### Hepatitis

The causative agent of hepatitis is a virus that infects the liver and causes swelling and inflammation. Several miRNAs namely miRNA-122, miRNA-340. miRNA-16 and miRNA-21 are being described to play a central role in hepatitis (121). Out of which miRNA-122 and miR-34a are considered as biomarkers of hepatitis-related hepatocellular carcinoma (HCC) while miRNA-21 is reported to be overexpressed in case of HCC (122). In the case of hepatic fibrosis, the expression of miR-21 is increased by TGF-β signaling whereas its decreased expression cause suppression of SMADT signaling. Higher expression of miR-34 and miRNA-122 is connected with secretion of TGF-β from hepatic cells, leading to the development of fibrosis. miRNA-122 particularly binds to the 5'end of the HCV gene, increase viral replication leading to the progression of the infection (123). miR-101 provides insights about the hepatitis-B surface antigen by their increased expression. The upregulation of miRNA-149, miRNA-638, and miRNA-491 enhance viral replication by inhibiting AKT/Pi3 pathway. Upregulation of miRNA-196 and miRNA- 448 is s observed in HCV-infected patients, which targets the coding region and NS5A of HCV genomic RNA. This deregulation is s associated with several signaling pathways, namely PI3K-Akt cascade, T-cell receptor pathway, mitogen-activated protein kinase (MAPK) signaling, viralcarcinogenesis, chemokines signaling, TGF-β signaling and Wnt signaling pathway (124).

### Tuberculosis

Pulmonary tuberculosis (TB) is an infection caused by *Mycobacterium tuberculosis*. In the majority of cases, the bacteria are in a dormant state before it grows into an active form. It most commonly affects the human lungs (pulmonary TB) but is also reported to affect lymph nodes, CNS, liver, bones, genitourinary tract and gastrointestinal tract (extrapulmonary TB) (125). Recent literature significantly displayed the link between tuberculosis and miRNAs. The cellular component and related gene expression are altered in patients suffering from tuberculosis. These changes are likely to be regulated by miRNAs. The existing literature reflect the role of several miRNAs in the differentiation and functioning of T-cells (126). Differential expression of miRNA-29c, miRNA-320, miRNA-101, miRNA-378, miRNA-483-5p, and miRNA-22 are diagnostic markers for tuberculosis and non-tuberculosis infections. Out of which, miRNA-378 and miRNA-101 are linked to MAPA1 signaling while miRNA-483-5p, miRNA-22, and miRNA-320 disturb BCL9L and AKT-3 signaling to initiate tuberculosis infection (127). Overexpression of miRNA-29 is observed in several human cell types in case of tuberculosis infection. This overexpression downregulates IFN-γ by acting on 3′UTR of IFN-γ mRNA. It formed an association of IFN-γ mRNA with Ago 2 protein forming a RISC, resulting in the post-transcriptional suppression of IFN-γ. miRNA-29 also have a key role in regulation of apoptotic pathways in immune cells by targeting myeloid cell leukemia-1 (Mcl-1), B-cell lymphoma 2 (Bcl-2) and the GTP binding protein Cdc42. Overexpression of miR-29, through some light on the mechanism of increased apoptosis in the cells involved in anti-tubercular response (125). The upregulation of miRNA-365 inhibits IL-6 signaling by binding to the 3′UTR region. Downregulated miRNA-155 corresponds to low TNF production via the mediation of the TLR-MAPK/AKT pathway. miRNA-155 degrades inositol phosphatase SH2 containing inositol-5-phosphatase (SHIP1) mRNA, therefore, increasing TNF production (127). In macrophages, the expression of miR-147 is activated by TLRs/NF-κB pathway, which further decreases the expression of TNF-α and IL-6. In serum or PBMCs of active tuberculosis patients, higher level of these inflammatory cytokines are recorded. Significant reduction in the growth of *M. tuberculosis* and significant increase in the expression of pro-inflammatory cytokines is observed when miR-99b is blocked using antagonists and knockdown approaches (125).

### Malaria

Malaria is a protozoal disease instigated by *Plasmodium vivax, P. faliciparum, P. malariae, P. knowlesi*, and *P. ovale*. This protozoal parasite potentially alters the expression of erythrocytic miRNA in blood. Downregulation of miRNA-451 and miRNA-16 have been seen in the blood/serum of malaria patients as compared to normal individuals. This downregulation is possibly due to the degradation of red blood cells and the clearance of miRNA in the case of hypersplenism during malarial infection, resulting in increased destruction of RBC by the spleen (128). In RBCs of malaria-infected patients, miRNA-451, miRNA-16, miRNA-106, miRNA-7b, miRNA-91, miRNA- 142, miRNA-144, let-7a, let-7f, and miRNA-92 are downregulated, whereas miRNA-19b and miRNA-223 are upregulated. miRNA-92 and miRNA-17 regulates TGF-β signaling, and are found to be responsible for renal failure by inducing apoptosis in the renal progenitor cells (129).

### Trypanosomiasis

Trypanosomiasis (sleeping sickness) is a protozoal infection disease. During early phase, trypanosomes activate the innate immune system and affects the B- and T-cell response. In trypanosome infected tissues, dramatic alteration in the macrophages and APCs have been observed. This results in the production of proinflammatory cytokines (like TNF-α, IL-6, and NO), mediated by variant surface glycoprotein (VSG). During the late phase, neurological manifestations along with high levels of TNF-α and inflammatory signatures have been widely reported (130). Significant reduction in the levels of miRNA (miRNA-199a-3p, miRNA-27b, and miRNA-126) have been reported in patients suffering from trypanosomiasis. These miRs deregulate toll-like receptor signaling and NF-κB cascade. Patients suffering from this disease revealed a higher level of miRNA-193b and miRNA-338 as compared to control ones (131). Downregulation of miRNA-27b has been observed in this disease. Increased levels of IFN- γ has been reported during trypanosomiasis infection. At the time of infection, the expression of miRNA-144 is decreased. miRNA-144 inhibits TNF-α and IFN-γ, suggesting the role of miRNA as a diagnostic marker for analyzing infection (132).

## Summary and Conclusion

In this review, we have discussed the brief outline of the role of miRNAs in immune regulation during infectious and inflammatory diseases. miRNAs are short RNAs, formed from the non-coding region of the RNA, which regulates the gene and protein expression by transcriptional inhibition. miRNAs are modulators of inflammatory and immune responses. Some miRNAs act as important inhibitors, while others tend to enhance the responses of the immune system by negatively regulating the response of the inhibitors. miRNAs either work on signal transduction proteins or support the inflammatory or anti-inflammatory responses of immune systems. Hence, miRNAs can act as biomarkers or targets for treatment in a variety of infectious diseases. Although miRNA- based therapy has limitations, further research is required to expand our knowledge of immuno-miRs. It can be considered as a futuristic approach for the diagnosis and treatment of immune-related diseases (acute and chronic inflammatory disorders) and infectious diseases.

## Author Contributions

KC and MG prepared the manuscript. MS supervised and corrected the manuscript.

### Conflict of Interest

The authors declare that the research was conducted in the absence of any commercial or financial relationships that could be construed as a potential conflict of interest.
